# Recurrence pattern analysis after [^68^Ga]-DOTATATE-PET/CT -planned radiotherapy of high-grade meningiomas

**DOI:** 10.1186/s13014-018-1056-4

**Published:** 2018-06-14

**Authors:** Barbara Zollner, Ute Ganswindt, Cornelius Maihöfer, Stefanie Corradini, Nathalie Lisa Albert, Christian Schichor, Claus Belka, Maximilian Niyazi

**Affiliations:** 1 Department of Radiation Oncology, University Hospital, LMU Munich, Marchioninistr. 15, 81377 Munich, Germany; 2 Department of Nuclear Medicine, University Hospital, LMU Munich, Marchioninistr. 15, 81377 Munich, Germany; 3 Department of Neurosurgery, University Hospital, LMU Munich, Marchioninistr. 15, 81377 Munich, Germany; 4 German Cancer Consortium (DKTK), partner site Munich; and German Cancer Research Center (DKFZ), Heidelberg, Germany

**Keywords:** Atypical and anaplastic meningioma, Radiotherapy, Recurrence pattern, Safety margin, IMRT

## Abstract

**Background:**

The aim of the present study was to evaluate the influence of the applied safety margins of modern intensity-modulated radiotherapy (IMRT) in patients with high-grade meningiomas on local control and recurrence patterns.

**Methods:**

Twenty patients with a neuropathological diagnosis of a high-grade meningioma (WHO°II or °III) treated with adjuvant or definitive radiotherapy between 2010 and 2015 were included in the present retrospective analysis. All patients were planned PET-based. Recurrence patterns were assessed by means of MRI and/or DOTATATE-PET/computertomography (CT).

**Results:**

The median follow-up was 31.0 months [95% confidence interval (CI): 20.1–42.0] and the progression-free survival (PFS) after 24 months was 87.5%. Overall, four patients had a local recurrence of their meningioma. Of these, three were located in field according to the prior radiotherapy treatment region, while only one patient had a distant relapse. There were no independent factors influencing progression-free or overall survival (OS).

**Conclusion:**

After radiotherapy (RT), patients with atypical or anaplastic meningiomas still have a defined risk of tumor recurrence. The aim of the present study was to examine mono-institutional data concerning target volume definition and recurrence patterns after radiotherapy of high-grade meningiomas as there are limited data available. Our data suggest that extended safety margins are necessary to achieve a favorable local control for high-grade meningiomas.

## Background

Meningiomas account for 20–30% of all primary intracranial neoplasms and represent the most common intracranial tumors in adults [1–4]. High-grade meningiomas show an excessive mitotic index on histopathological examination [5]. Additional criteria for the diagnosis of atypical meningiomas are brain invasion or three of the five following histopathological aspects: prominent nucleoli, high cellularity, small cells, spontaneous necrosis or sheeting, i.e. loss of whorling or fascicular architecture [6, 7]. Overall, high-grade meningiomas (WHO (World Health Organisation) °II and °III) show a significantly more aggressive behavior and poorer outcome as compared to low-grade meningiomas [8–14].

A multimodal treatment approach with a combination of surgery and radiotherapy (RT) is nowadays considered to be the treatment of choice [8, 15]. Due to the frequent adhesion to neurological structures such as the optical nerve, optical chiasm or brainstem, gross total resections remain challenging [14]. Therefore, postoperative RT is recommended for most cases of atypical meningiomas WHO grade II and all anaplastic meningiomas WHO grade III. A large multicenter analysis with more than 2000 patients by Wang et al. concludes that adjuvant radiotherapy after subtotal resection of an adjuvant meningioma significantly improves the overall survival while it does not after gross total resection [16]. As Condra et al. pointed out, high-grade meningiomas have a poorer outcome as compared to low-grade meningiomas and benefit from a more aggressive treatment including postoperative radiotherapy [17], as RT is known to significantly improve local control [12]. Over the past decades, radiotherapy techniques improved substantially, as new modalities such as intensity modulated radiotherapy (IMRT) or the image-guided application of radiotherapy (IGRT) using robotic patient positioning couches increasing the precision and accuracy of radiotherapy [18]. Thus, radiotherapy has become a more valuable treatment option in the management of high-grade meningiomas.

While the indication for postoperative RT in high-grade meningiomas appears undoubted, the appropriate target volume definition and radiation dose remain a matter of debate. We present a mono-institutional retrospective analysis on the recurrence patterns of high-grade meningiomas after RT with a special emphasis on radiation dose and safety margins of target volumes.

## Materials and methods

### Patient selection

Patients with histopathologically proven atypical or anaplastic meningioma who underwent RT at our department from 05/2010 to 09/2015 were included in this retrospective study. Even if patients did not have prior surgery within 12 weeks before radiotherapy, there was a former histopathological result confirming a high-grade meningioma. Patients were excluded if they had prior radiosurgical treatment or EBRT at the same site. Altogether 20 patients with atypical or anaplastic meningioma were included in this analysis. All of them were initially PET-positive and therefore were planned DOTATATE-based. Fourteen patients had prior surgery, while six patients were treated with definitive radiotherapy. Patients’ and tumor characteristics are shown in Table 1.

**Table 1:** Patient characteristics, *n* = 20 (PTV_hom_ = PTV homogenous, PTV_ext_ = PTV_extended_, PTV_boost_ = PTV simultaneous integrated boost, CTV_hom_ = CTV homogenous, CTV_ext_ = CTV extended)

Characteristic	Patients
Sex	
Male	14 (70%)
Female	6 (30%)
Median age (range)	61 years (26–79)
Age < 50 years	6 (30%)
Median follow-up [months], 95%-CI	31.0 (20.1–42)
Surgery pre-RT	14 (70%)
Gross total resection	10 (50%)
Subtotal	3 (15%)
Debulking	1 (5%)
Simpson grade of resection	
I	9 (45%)
II	1 (5%)
III	0
IV	3 (15%)
V	1 (5%)
Recurrence patterns	
No recurrence	16 (80%)
In-field recurrence	3 (15%)
Marginal recurrence	0
Ex-field recurrence	1 (5%)
Median dose of RT [Gy]	60 (59.4–60.0)
Median interval between PET-scan and RT [months]	1.3 (0–9)
WHO grade	
II	16 (80%)
III	4 (20%)
Localization	
Frontal	14 (70%)
Frontoparietal	3 (15%)
Frontotemporal	1 (5%)
Parietooccipital/occipital	2 (10%)
Technique	
Step-and-shoot-IMRT (# SIB)	18 (90%)/6 (SIB)
3D	2 (10%)
Median safety margin GTV → CTV_hom_ or CTV_ext_	15 mm (2–20 mm)
Median safety margin CTV → PTV_hom_ or PTV_ext_ [mm]	4 (2–7)
Median safety margin GTV → PTV_boost_ [mm]	3 (0–10)
Mean GTV size [ml]	64.5 (14.8–192.7)
Mean PTV size [ml]	
PTV_ext_ or PTV_hom_	301.5 (83.7–743.0)
PTV_boost_	190.3 (20.5–586.2)

All patients gave their written informed consent for the treatment. This retrospective analysis was approved by the ethics committee of the LMU Munich on record number 545–16. There was no experimental research on humans or animals performed or reported. The declaration of Helsinki has been obeyed in all points.

### Treatment and follow-up

A [^68^Ga]-DOTATATE-PET/CT and a Gadolinium-enhanced magnetic resonance imaging (MRI) of the brain were performed and fused with the treatment planning CT to delineate the target volume. To ensure reproducibility patients were immobilized with a thermoplastic mask system. For critically located lesions, a double-layered thermoplastic mask system was used to minimize setup uncertainties. Treatment planning was performed using the Oncentra® treatment planning system (OTP MasterPlan®, Elekta, Crawley, UK) for 3D-conformal RT and Hyperion® for IMRT which employs constrained optimization using a Monte Carlo dose algorithm [19]. Intensity-modulated radiotherapy (IMRT) was used if adjacent critical organs at risk structures were present. Organs at risk (OAR) constraints were chosen according to conservative estimates given by QUANTEC [20] - the constraints for maximum point doses were 54Gy for optic pathway structures including optic chiasm [21] and 54 Gy for the brainstem [22]. The mean cochlear dose was optimized to be lower than 45 Gy. Planning target volume (PTV) was defined as gross tumor volume (GTV) for patients with macroscopic tumor or resection cavity for patients with prior surgery plus a 15 mm isotropic margin for clinical target volume (CTV) with an additional 3–5 mm PTV expansion. Individual adaptions were made and are shown in Table 1. GTV included the contrast enhancing lesion in T1w + Gd MRI and was adapted to the DOTATATE-enhancement to detect tumoral dural tails or bone infiltration. All patients with prior resection were planned on the resection cavity,

The outcome was evaluated on a regular basis (first time three months after RT, later once per year) using an MRI of the brain and/or DOTATATE-PET-CT in case of suspicious MRI findings. Similarly to the study of Lee et al. recurrence of the tumor was defined as “in-field” if more than 80% of the tumor were located within the prescribed 95%-isodose [23]. A “marginal” recurrence was present if 20–80% were inside the 95%-isodose surface. And any other recurrence was defined as “ex-field”. If there was multifocal recurrence, the tumor volume that was most distant to the initial tumor site was taken as a reference.

### Statistics

Overall survival (OS) as well as progression-free survival (PFS) and local progression-free survival (LPFS) were measured from the beginning of RT to progression, death or respectively the date of last follow-up. For the latter, patients who died because of medical co-morbidities or had a distant relapse were censored. The Kaplan-Meier method was used for survival analysis. 95% confidence intervals (CI) were calculated using the associated estimated standard errors. Survival estimates were compared using the log-rank test. A Cox regression analysis was performed to identify factors influencing overall survival or progression-free survival. *P*-values were considered as significant at ≤0.05.

## Results

A summary of baseline patient, tumor and treatment characteristics is shown in Table 1. Median age was 59.7 years (range 26–79 years). Sixteen patients had a meningioma grade II and four patients had a meningioma grade III. Fourteen patients underwent surgery before RT (within ≤12 weeks). Four patients had a relevant time delay to the start of RT due to various reasons (refusal, comorbidities). Two patients had no surgery prior to RT, as the tumor volume was too extensive. Surgery resulted in a gross total resection in 10 cases, subtotal in three and a debulking resection in one case. Grading for the extent of tumor resection was determined according to the study of Simpson et al. [24]. Nine patients had a Simpson-grade I resection, one patient a grade II resection, three had a grade IV resection and one had a Simpson-grade V resection. Nine of the patients having surgery within 12 weeks before radiotherapy had undergone at least one other previous resection before the surgery that led to radiotherapy. Tumor localization was frontal (70%), frontoparietal (15%), frontotemporal (5%), occipital (5%) and parietooccipital (5%).

**Fig. 1:**
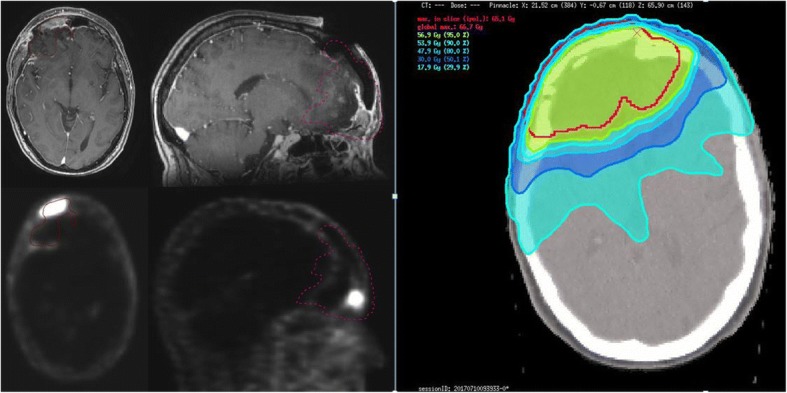
Example of a patient with a right frontal meningioma s/p subtotal resection while postoperative MRI does not unequivocally show a tumor residual but radiologists describe postoperative granulation tissue. The PET/CT scan nicely visualizes the bone involvement, on the right the IMRT plan including PTV and 95%/90%/80%/50%/30%-isodoses, respectively

Concerning radiotherapy, median number of fractions was 30 (28–33) with a median dose per fraction of 2.0 Gy (1.8–2.14 Gy) and a median total radiation dose of 60.0 Gy (59.4–60.0 Gy) (Table 1). Moderate hypofractionation (single dose 2.14 Gy) was only exceptionally used as simultaneous integrated boost (SIB) dose in two cases (59.92 Gy cumulative SIB dose). All patients had a pre-radiotherapeutic [^68^Ga]-DOTATATE-PET-CT. Mean tumor maximum standardized uptake volume (SUV_max)_ was 9.76 (0–25.3). Fig. 1 shows a patient with a right frontal meningioma where the additional [^68^Ga]-DOTATATE-PET-CT shows an impressive infiltration of the skull base which was not unequivocally visualized by postoperative MRI.

The median cumulative margin from gross tumor volume (GTV) to planning target volume (PTV) was 20 mm at maximum, in median 15 mm to generate the CTV and additional 4 mm in median for PTV. In five patients, the pre-operative volume was mainly used to define the adapted clinical target volume, in all other patients either remaining macroscopic tumor or the resection cavity was used. Six patients had a concept with a simultaneous integrated boost (SIB). We considered the high-dose volume as the PTV_boost_ in case of a SIB and the surrounding PTV-volume as PTV_extended_ (PTV_ext_). In cases without SIB the PTV was named PTV_homogeneous_ (PTV_hom_). For PTV_boost_ the GTV was expanded 3 mm in median.

Four of the 20 patients had a relapse of their meningioma. Regarding recurrence patterns, two patients (10%) with a recurrence after radiotherapy had an in-field recurrence concerning the PTV_hom_. One patient (5%) had radiotherapy with a simultaneous integrated boost concept whose recurrence was located in field of the PTV_ext_ but a marginal recurrence regarding the PTV_boost_. Another patient developed a distant relapse of the tumor, which may be considered as a second meningioma without any relation to the primary lesion. Regarding the in-field relapses, all patients had macroscopic tumor at the time of RT: in one case, there was a debulking resection prior to radiotherapy, in another case there was a relapse of the tumor after surgery and in the third case, there was no resection possible, as the tumor, volume was considered too large. One recurrence occurred in a patient with an anaplastic meningioma, the other three occurred in patients with atypical meningioma. There is no statistical significance, which is due to the small number of only four patients having a recurrence. In two cases, recurrence was diagnosed by MRI and DOTATATE-PET/CT. In the two other cases, recurrences were also diagnosed by MRT and PET, but surgery was performed afterwards so that there is also a histopathological proof for the recurrence.

**Fig. 2:**
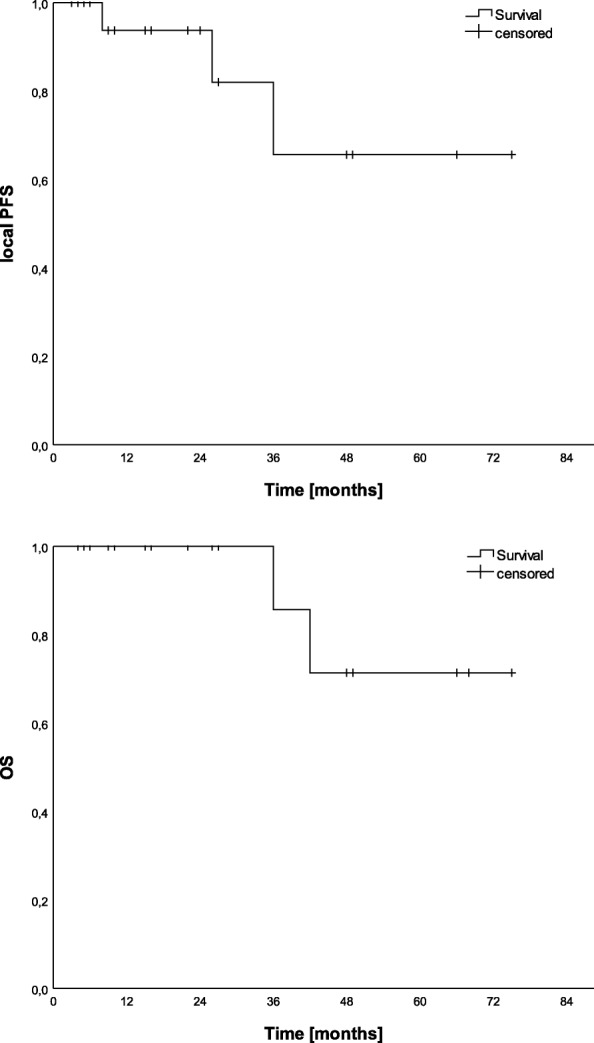
Local progression-free survival (LPFS) and overall survival (OS) [Kaplan-Meier method]

Median follow-up was 31.0 months [95%-CI: 20.1–42.0]. Median overall survival was 64.7 months [95%-CI: 52.6–76.8] (Fig. 2). Two patients died shortly after treatment, one of them had a relapse of the tumor. Both deaths were not related to the meningioma. Local progression free survival was 87.5% after 24 months and 70% after 36 months (Fig. 2).

Patients presenting with a local recurrence had a median margin from GTV to CTV of 12.5 mm plus another 4 mm margin for PTV (either PTV_ext_ or PTV_hom_). In one patient with a local relapse who had a SIB concept, the GTV was planned as boost volume without any additional safety margin.

In one case with a recurrence, there were relatively small safety margins from GTV to PTV (5 mm in total), the other three patients presenting with local relapse adequate margins of at least 15 mm were identified. Therefore, the detected tumor relapses would not have been avoided by using larger margins. Only in the case of the SIB-concept, a larger safety margin to the PTV_boost_ would have been advantageous but was not possible because of the surrounding organs at risk (optical system).

Overall, radiotherapeutic toxicity was low. Despite mild radiodermatitis Grade I-II (*n* = 9), there were no higher graded toxicities such as radiation necrosis. One patient suffered from a transitory severe hyperglycemia associated to steroid medication given due to increased intracranial pressure.

On univariate analysis for categorical variables (Table 2), no significant factors influencing OS or PFS were identified (age < 50 years, WHO grade, SIB or non-SIB-concept, type of resection (total vs. subtotal/biopsy), histology or Simpson grade of resection). For continuous variables such as age, SUV_max_, time between DOTATATE-PET and treatment beginning, timing of RT (post-operative versus salvage), size of the PTV_hom/ext_ no influence on PFS or OS could be detected as well. All patients were DOTATATE-planned and there was a slight trend that patients with a high SUV_max_ were more likely to have a relapse of the tumor reflecting macroscopic tumor at the beginning of the treatment.

**Table 2:** Cox regression analysis on potentially prognostic factors and their impact on overall and progression-free survival, *n* = 20, HR = hazard ratio

Variable	HR (Univariate *p*-value)	
	OS	PFS
Age	1.24 (0.31)	1.04 (0.40)
Sex	0.00 (0.81)	0.55 (0.63)
PTV_hom/extended_ volume	0.00 (0.38)	0.03 (0.35)
SIB vs. Non-SIB	0.03 (0.58)	0.6 (0.66)
Size of PTV	1.00 (0.63)	1.00 (0.40)
Simpson grade	0.25 (0.58)	1.06 (0.90)
Histology (°II vs. °III)	2.24 (0.57)	1.82 (0.61)
Type of resection (total vs. subtotal/biopsy)	0.01 (0.58)	0.92 (0.90)
Timing of RT (immediately vs. after ≥1 recurrence)	3.54 (0.38)	4.4 (0.22)
SUV_max_ (DOTATATE-PET)	0.98 (0.88)	1.15 (0.09)

## Discussion

The aim of the present study was to examine mono-institutional data concerning target volume definition and recurrence patterns after radiotherapy of high-grade meningiomas as there are limited data available. Table 3 shows an overview of the existing data in comparison to our study.

**Table 3:** Dose and Safety Margins (*excessive mitotic figures without other features of frank malignancy)

Author	Number of cases (patients with RT)	WHO Grade	Mean Dose (if not otherwise specified) [Gy]	CTV-Margin [mm]	PTV-Margin [mm]	Local control rate (at respective timepoint)
Aboukais et al. [40]	167 (27)	II	53.8	10–20	5	Median PFS 8.2y
Hug et al.[41]	31 (31)	II + III	58	Not mentioned	Not mentioned	46.5% (no time mentioned)
Park et al.[42]	83 (27)	II	median61.2	15	3	58.7% (no time mentioned)
Kumar et al. [14]	37 (37)	II + III	54	10–20	5	58% [°II]; 20% [°III] (5y)
Boskos et al.[43]	24 (24)	II + III	65	5–20	Not mentioned	46.7% (8y)
Aghi et al.[44]	108 (38)	II	60.2	*10 in total*		100% (3.1y)
Dziuk et al.[45]	48 (19)	III	54	*30–40 in total*		25% (5y)
Press et al. [35]	54 (54)	II	59.4	5	3	74% (3y)
Choi et al. [29]	114 (89)	II + III	60	10–20	Not mentioned	68% (5y)
Condra et al. [16]	262 (21 (S + RT) / 7(RT alone))	I-III	53.3 (post-op) / 51.7 (RT alone)	*20 in total*		78% (15y) / 86% (5y)
Glaholm et al.[46]	186 (43)	II + III and aggressive I*	Range 50–55	Not mentioned	Not mentioned	78% (5y)
Milosevic et al. [28]	59 (59)	II + III	50	*30–40 in total*		34% (no time mentioned)
Goldsmith et al. [31]	140 (140, 23 of malign type)	I-III	54	*10–30 in total*		89% (5y)
Goyal et al. [27]	22 (8)	II	54	Not mentioned	Not mentioned	71% (5y)
Adeberg et al.[47]	85 (84)	II + III	57.6	10–20	1–5	50% [°II]; 13% [°III] (5y)
Engenhart-Cabillic [48] et al.	16 (7)	II + III	55.5–60	Not mentioned	Not mentioned	62.5% (2.3y)
Pasquier et al.[49]	119 (119)	II + III	54.6	Not mentioned	Not mentioned	62% [°II]; 48% [°III] (5y)
Katz et al. [32]	36 (36)	II + III	55–60	Not mentioned	Not mentioned	45% (5y)
Present study	20	II + III	59.8	15	4	LPFS 87.5% (24 months)

Compared to the existing literature this analysis shows a favorable local control rate for high-grade meningioma after radiotherapy with a total dose of 60 Gy and a safety margin of 15 mm for CTV and additional 4 mm for PTV. In the present analysis, most recurrences were observed in field in patients with macroscopic tumors. A local dose escalation could probably improve the local control rates and should be evaluated in further studies. In comparison with other studies we could not identify factors which influenced overall or progression-free survival.

In contrast to other studies, all treatments of the present analysis were planned by using an MRI and a [^68^Ga]-DOTATATE-PET/CT fusion. Usually, target delineation is mainly based on contrast-enhanced MRIs (pre- and postoperative) only [25, 26]. Additional [^68^Ga]-DOTATATE-PET/CT scanning can provide valuable information on bone infiltration or dural tails [27].

Goyal et al. noticed that atypical meningioma benefit from gross total resection (Simpson Grades I-III), as gross total resection is associated with better local control rates, but he concludes that the role of postoperative RT remains unclear [28]. In contrast, Milosevic et al. recommend immediate RT after initial surgery for high-grade meningiomas [29]. Choi et al. conclude with the fact that postoperative radiotherapy could improve local control in patients with high-grade meningiomas after incomplete surgical resection and emphasize that a gross total resection is the most important factor for local control [30]. In contrast to these studies, Champeaux et al. failed to demonstrate a significant improvement in different clinical outcomes after RT for meningioma grade II [31]. We could not identify a difference between patients treated post-operative or salvage, which is likely due to our small number of patients.

Goldsmith et al. suggested a radiation dose of 60 Gy for high-grade meningiomas [32]. This suggestion is based on their retrospective analysis of 140 patients whereas 23 of them had a malignant type of meningioma and were treated in median with 54 Gy (range 44.62 to 69.26Gy) [32]. Katz et al. performed an analysis with an accelerated hyperfractionated RT for patients with atypical and anaplastic meningioma [33]. Thirty-six patients were treated with 60 Gy in 1.5 Gy single dose twice per day [33]. Local control rate was significantly poorer with 45% in comparison to less aggressive treatment schedules but caused significantly higher toxicity [33]. This study concluded that 50–60 Gy delivered with once-daily fractionation seems feasible as it looks unlikely that more aggressive RT could improve outcome [33]. Combs et al. suggest on the one hand that PTV has to be enlarged to the resection cavity plus a safety margin of 1–2 cm and on the other hand advocate for a dose escalation to 60–66 Gy [8].

The results of the EORTC-trial 22,042–26,042 are eagerly awaited and will highlight the role of dose escalation in this setting. In this trial, patients with Simpson grades 1 to 3 receive standard postoperative RT, while patients with a higher grade receive an additional boost. There are also few data indicating that hypofractionation might be an effective option. Maranzano et al. describe their long-term results of moderate hypofractionated stereotactic radiotherapy for intracranial meningiomas [34]. In this study 77 patients were treated with a median volume of 23 cm^3^ while dose was prescribed either in 15 × 3 Gy or 14 × 3 Gy [34]. The authors conclude that moderate hypofractionation had a good outcome and was tolerated well [34] whereas a longer follow up and further trials should be performed to corroborate these findings.

However, any kind of dose escalation or hypofractionation should be done carefully, considering the adjacent organs at risk and their dose tolerance. Bostrom et al. report about a patient developing severe radiation necrosis due to the RT in several lesions (some of them treated hypofractionated) [35].

Considering the planning process and the safety margins an analysis by Press et al. found that meningioma grade II treated with conformal IMRT and limited safety margins < 1 cm, did not lead to a higher rate of recurrence [36]. Six of 46 patients had a tumor relapse [36]. The study confirms the findings of the present analysis, as most relapses occurred “in-field”. Press et al. found 5 of 6 recurrences within the prior radiation volume and one was combined in-field and marginal [36]. A safety margin of 5 mm for CTV and 3 mm for PTV was applied in this patient cohort [36]. Press et al. postulate that as none of their patients relapsed marginally safety margins of < 1 cm might be sufficient [36]. In contrast to the present study, the majority of patients did not have macroscopic tumor. Taken together, the margins for grade II meningiomas cannot be transferred unreflectedly to meningiomas grade III. Regarding the existing studies a limited safety margin might be an option for some atypical meningiomas grade II, but for anaplastic meningiomas grade III a limited safety margin might be harmful and could lead to tumor relapse. The present data suggest that safety margins for anaplastic meningiomas have to be sufficiently large.

Over the last decades, modern radiotherapy techniques (IMRT or volumetric modulated arc therapy (VMAT)) were introduced in the treatment of meningiomas. Anvari et al. conclude that utilizing high-technology equipment and new techniques might improve the outcome after RT [37].

Madani et al. describe that dose-painting intensity-modulated proton therapy (IMPT) using a SIB is a feasible therapy option with excellent dose coverage with minimal or no dose to brain, brainstem or optical system [38]. A study by Simon concludes that carbon-ion RT could be the preferred therapy option [39]. Similarly, Harrabi et al. describe that proton beam RT shows dosimetric advantages over conventional radiotherapy that might be essential for neurologic function [40]. Therefore, future concepts might include carbon-ion or proton RT.

The present study is limited due to its retrospective nature and the small number of patients. Obviously the number of or presented patients is smaller than in most of the mentioned studies in Table 3. In contrast you have to consider that in the other mentioned studies, there are all kind of meningioma, not only high-grade meningiomas as presented here.

The follow-up time has a wide range, as some patients had to be censored very early. Furthermore, a multivariate analysis was not reasonably feasible due to the small number of cases, which is another weakness of the analysis.

## Conclusion

Even following postoperative radiotherapy, high-grade meningiomas relapsed frequently “in-field” of the prior target volume.

We consider postoperative radiotherapy after resection of a high-grade meningioma essential to minimize the risk of local failure, even if a gross total resection of the tumor was performed. Especially patients with macroscopic tumor should receive postoperative RT immediately. Patients who cannot be treated with a resection of the tumor at all should receive a definitive radiotherapy.

Concerning target volume delineation, the present data show a good local control with the applied margins and confirm the common used safety margins. We suggest a CTV margin of 15 mm starting from the GTV and additional 3–5 mm for the PTV, depending on the available image-guidance.

It would be valuable to have future studies including a higher number of patients to evaluate which patients would benefit from a dose escalation, e.g. in form of a simultaneous integrated boost, or a CTV margin reduction.

## Abbreviations

CI: Confidence interval
CT: Computer tomography
CTV: Clinical target volume
GTV: Gross tumor volume
IGRT: Image-guided application of radiotherapy
IMRT: Intensity modulated radiotherapy
LPFS: Local progression-free survival
MRI: Magnetic resonance imaging
OAR: Organs at risk
OS: Overall survival
PFS: Progression-free survival
PTV: Planning target volume
PTVext: Planning target volume extended
PTVhom: Planning target volume homogenous
RT: Radiotherapy
SIB: Simultaneous integrated boost
SUVmax: Maximum standardized uptake value
VMAT: Volumetric modulated arc therapy
WHO: World Health Organisation
